# Isolation and Characterization of Cellulose Nanocrystals from Bacterial Cellulose Synthesized via *Ancylobacter* sp. STN1A Using Residual Glycerol

**DOI:** 10.3390/polym17091240

**Published:** 2025-05-01

**Authors:** Manuel Peña-Ortiz, Araceli García, Sophie Marie Martirani-Von Abercron, Patricia Marín, Silvia Marqués, Ramzi Khiari, Alain Dufresne, Luis Serrano

**Affiliations:** 1 Nanoval FQM-383 Research Group, Organic Chemistry Department, University of Córdoba, Marie Curie (C-3) Building, Ctra. Nnal. Km 396, 14014 Córdoba, Spain; b52penom@uco.es (M.P.-O.); qo2ganua@uco.es (A.G.); 2 BioPrEn RNM 940 Research Group, Inorganic Chemistry and Chemical Engineering Department, University of Córdoba, Marie Curie (C-3) Building, Ctra. Nnal. Km 396, 14014 Córdoba, Spain; 3 Faculty of Science, Instituto Químico Para la Energía y el Medioambiente (IQUEMA), University of Córdoba, Marie Curie (C-3) Building, Ctra. Nnal. Km 396, 14014 Córdoba, Spain; 4 Estación Experimental del Zaidín, Department of Biotechnology and Environmental Protection, Consejo Superior de Investigaciones Científicas, C/. Profesor Albareda 1, 18088 Granada, Spain; sophie.martirani@eez.csic.es (S.M.M.-V.A.); pmarin@eez.csic.es (P.M.); silvia.marques@eez.csic.es (S.M.); 5 Department of Textile, Higher Institute of Technological Studies of Ksar Hellal, Ksar Hellal 5070, Tunisia; khiari_ramzi2000@yahoo.fr; 6 Centre National de la Recherche Scientifique (CNRS), Grenoble INP, Laboratory of Process Engineering for Biorefinery, Bio-Based Materials and Functional Printing (LGP2), Université Grenoble Alpes, F-38000 Grenoble, France; alain.dufresne@pagora.grenoble-inp.fr

**Keywords:** bacterial nanocellulose, *Ancylobacter*, crude glycerol, nanocrystal, characterization

## Abstract

Given the growing interest in the functional properties of nanocellulosic forms, bacterial cellulose nanocrystals (BCNCs) have gained attention as sustainable, high-performance materials for diverse applications. Although recent research has addressed the use of agro-industrial waste for BCNCs production, limited attention has been given to residual crude glycerol, a widespread byproduct of the biodiesel industry. Therefore, this work aimed to synthesize and thoroughly characterize BCNCs from bacterial nanocellulose (BNC) obtained through the metabolism of crude glycerol via the novel bacterial strain *Ancylobacter* sp. STN1A. The influence of sulfuric acid (H_2_SO_4_) hydrolysis time on BCNCs´ morphology and physicochemical properties was evaluated. Severe hydrolysis conditions yielded shorter, narrower nanocrystals (0.91 μm × 40 nm; L/D = 22.8) with increased crystallinity (63%) and high colloidal stability (−40.17 ± 0.68 mV), as well as slightly reduced thermal stability. In contrast, milder conditions produced longer BCNCs (1.13 μm × 42 nm; L/D = 26.9) with similarly high zeta potential (−44.13 ± 0.73 mV), while maintaining the thermal and crystalline features of the starting BNC. These findings demonstrate the potential to tailor BCNCs´ properties through controlled hydrolysis and support the viability of producing versatile nanocellulosic materials from residual byproducts, contributing to both cost-effective production and environmental sustainability.

## 1. Introduction

Cellulose, a linear homopolymer made up of D-glucopyranose units linked by β-1,4-glycosidic bonds, is the most abundant organic compound in the biosphere [1]. Although mainly present in plant cell walls, cellulose can also be found in certain species of algae, marine tunicates, and bacteria, a fact that deeply affects its specific structural organization [2]. This widespread presence makes cellulose a virtually unlimited resource, capable of addressing the growing demand for environmentally sustainable and biocompatible materials [3].

Over recent years, there has been a growing interest in utilizing cellulose as a nanostructured material, particularly in the form of nanofibrillated cellulose (NFC) and cellulose nanocrystals (CNCs) [4]. These nanostructures, generally referred to as nanocelluloses, exhibit at least one dimension in the nanoscale (<100 nm), thereby combining the inherent properties of cellulose (renewability, low toxicity, hydrophilicity, biodegradability, and low density, etc.) with specific features on the nanoscale (high aspect ratio, improved mechanical strength, or high specific surface area, etc.). Consequently, these nanomaterials have recently been applied in diverse sectors, including biomedicine, food, automotives, aerospace, and cosmetics [5].

Regarding CNCs production, acid hydrolysis is the main ‘top-down’ method for deconstructing cellulose fibers, due to its technical and economic competitiveness over other extraction techniques [6]. During this process, hydronium ions penetrate the amorphous domains of cellulose chains, leading to the cleavage of β-1,4-glycosidic bonds and the subsequent release of crystallites [7]. Among the most commonly used acids for this conversion, sulfuric acid (H_2_SO_4_) is widely employed due to its shorter reaction time, higher conversion rate to CNCs, and ability to form stable colloidal systems [8]. Indeed, previous research has shown that the disruption of cellulose fibers via H_2_SO_4_ under varying times, temperatures, and acid concentration conditions promotes the generation of tailored CNC [9,10]. These nanocellulosic forms have been shown to exhibit high aspect ratios (>100) and remarkable mechanical properties [8,11].

The choice of the cellulose source for synthesizing CNCs is also a determining factor in shaping their morphology and properties [12]. Over the years, plants have mainly supplied the raw material for this purpose. This is attributed to the growing utilization of agricultural residues, aligned with the principles of the biorefinery approach within the pursuit of a circular economy. Some examples of materials commonly employed in this regard include garlic straw, sugarcane bagasse, and corncob [13]. However, the high energy requirements involved in processing agricultural residues have led to concerns about sustainability, prompting the search for new cellulose fiber sources [14].

Bacterial nanocellulose (BNC) has emerged as an appealing alternative due to its exceptional purity, remarkable crystallinity, biocompatibility, and biodegradability [14,15]. Although many bacterial taxa have the genetic potential for BNC synthesis, current production is mainly carried out by Gram-negative, slightly acidic, and aerobic species such as *Komagataeibacter xylinus* [16]. The production yield largely depends on the glucose derived from the carbon source during bacterial metabolism. Consequently, synthetic media enriched with carbon sources are extensively employed, causing a significant impact on the overall cost of BNC production to date [17]. In response to this economic challenge, recent research has focused on the isolation and genetic modification of bacterial strains capable of using agro-industrial residues and other byproducts [17,18]. These sources present a dual advantage, offering not only cost-effective solutions, but also environmentally sustainable approaches for BNC synthesis.

Crude glycerol, a common byproduct of biodiesel production, emerges as a promising source of industrial waste for this purpose. Generated at approximately 10% (*v*/*v*) during conventional transesterification processes, its high contaminant content restricts direct commercialization. As the biodiesel industry continues to grow, the accumulation of this residue is becoming an increasing environmental concern [19,20]. As a potential valorization route, newly described bacterial strains from the genus *Ancylobacter* have shown the ability to use crude glycerol as a carbon source for BNC production [16]. This approach could improve the economic viability of the biodiesel industry while reducing the overall cost of BNC production, thereby facilitating its further processing into bacterial cellulose nanocrystals (BCNCs).

To date, most studies on BCNCs production have involved a narrow range of bacterial strains grown in commercially available media [21,22,23]. From a waste valorization perspective, research efforts have primarily focused on agro-industrial residues such as apple and pineapple [24,25,26]. Nevertheless, a significant knowledge gap persists concerning the production and in-depth characterization of BCNCs derived from industrial crude glycerol. In this regard, Efthymiou et al. (2022) [27] reported the production of BCNCs through the fermentation of a crude glycerol-based medium supplemented with sunflower meal-derived proteins via *Komagataeibacter sucrofermentans*. However, their research mainly addressed the effect of incorporating these nanocellulosic forms into food packaging materials [27].

Given this situation, this work aimed to present an innovative approach for BCNCs production by utilizing residual crude glycerol as a substrate for BNC synthesis. For this purpose, the previously unexplored bacterial strain *Ancylobacter* sp. STN1A was employed for the first time, offering potential new opportunities for sustainable BNC production. Considering the influence of the previously discussed parameters on the final characteristics of BCNCs, we assessed the effect of the total acid hydrolysis reaction time on the morphology, surface charge, crystallinity, chemical structure, and thermal stability of the resulting nanocellulosic forms. Therefore, this study contributes new insights to the scientific literature on optimizing the properties of novel BCNCs types, emphasizing their potential applications.

## 2. Materials and Methods

### 2.1. Materials

Used cooking oil-derived crude glycerine (UCO-Glycerine) was obtained from the BioOils Huelva oil production plant (La Rábida, Spain) as a residue from advanced biodiesel production during the transesterification reaction of a mixture of used cooking oils, animal fat, and other residual oils. Sodium hydroxide (NaOH, CAS Number 1310-73-2, pellets, Reag. USP, for analysis, ACS, ISO, Panreac, Spain) was used for BNC purification. The concentration of both BNC and BCNCs was determined using Rotilabo^®^ aluminum sample bowls (125 mL, Carl Roth, Germany). To synthesize and obtain BCNCs, sulfuric acid (H_2_SO_4_, CAS Number 7664-93-9, 95–98%, ACS reagent, Sigma-Aldrich, St. Louis, USA) and dialysis membranes (Spectra/Por^®^ 1, molecular weight exclusion limit of 6–8 kD, Spectrum Laboratories, Rancho Dominguez, USA) were employed. For BCNC characterization, plastic cells (DTS0012) and disposable folded capillary cells (DTS1070) were purchased from Malvern Panalytical (Malvern, Worcestershire, UK).

### 2.2. BNC Synthesis and Purification

Bacterial cellulose was obtained by culturing *Ancylobacter* sp. strain STN1A in Widdel production medium (WPM), which had a similar composition to that of Widdel mineral medium (WMM), except that it contained 150 μL of vitamin solution, 300 μL SL-10 solution, and 200 μL of selenium–wolfram solution per 100 mL of medium [16]. The WPM was supplemented with unknown UCO-Glycerine to a final concentration of 2%. The cells were cultivated for 6 weeks under permanent darkness at 30 °C in sterile conditions, using stacked 20 × 20 cm Petri dishes filled with 200 mL of medium. Then, the cellulose biofilms were collected, rinsed with deionized water, and subjected to a purification process to remove bacterial cells and other components. To achieve this, the biofilms were rinsed with distilled water and treated with 0.1 N NaOH at 100 °C for 2.5 h. After this treatment, they were thoroughly washed with deionized water until neutral pH [28]. Finally, an Ultra-Turrax IKA T18 device was employed to disintegrate the biofilms (9000 rpm, 15 min), collecting five different samples of the resulting suspension to determine the average fiber concentration after oven-drying at 105 °C for 24 h [29]. The remaining dispersion was stored at 4 °C until use. The nomenclature assigned to this BNC suspension was ‘UCO-Gly’.

### 2.3. Preparation of BCNCs

For the preparation of BCNCs, previously described methodologies were followed with slight modifications [14,30]. Two different treatments were carried out to evaluate their influence on the properties of the resulting BCNCs. The first treatment, termed ‘BCNC-1’, was an acid hydrolysis with 50% (*w*/*w*) H_2_SO_4_ at 60 °C for 60 min, maintaining a BNC–reaction medium ratio of 1:110 (*w*/*v*). In the second treatment, called ‘BCNC-2’, the fibers were subjected to the same acid hydrolysis conditions in terms of acid concentration and temperature, but the total reaction time was reduced to 30 min. Based on the difference in duration, the treatment applied to BCNC-1 was considered a ‘severe’ condition, while BCNC-2 was deemed as ‘mild’. For each acid hydrolysis, 1 g of previously homogenized UCO-Gly suspension (Ultra-Turrax IKA T25 device, 9000 rpm, 4 min) was poured into a beaker. Then, each beaker was placed in a crystallizer filled with ice, ensuring that the dispersion was fully surrounded. Afterward, H_2_SO_4_ was cautiously added dropwise from a burette while maintaining constant and vigorous stirring throughout the reaction. Upon the completion of the acid addition, the temperature of the reaction medium was increased to the selected target temperature, using a heating plate coupled with a thermocouple. To stop the reaction, 10 times the reaction volume of cold distilled water was added. The resulting dispersion was subjected to centrifugation (11,000 rpm, 4 °C, 40 min) in a Sigma 3–18 KS centrifuge (Osterode am Harz, Germany), seeking to precipitate BCNCs and remove any excess acidic solution, a process that was repeated 3 times. Subsequently, both BCNC suspensions underwent ultrasonication (Branson Sonifier 250, Emerson Electric, St. Louis, MO, USA) for 7 min at a duty cycle of 60% and a micro tip limit of 5, ensuring their complete dispersion before being introduced into dialysis membranes. During this final stage, BCNC dispersions were kept submerged in distilled water until reaching a neutral pH. Finally, the final concentration was determined using the gravimetric method.

### 2.4. Morphological, Physicochemical, Spectroscopic, and Thermal Characterization

#### 2.4.1. Scanning Electron Microscopy (SEM) of BCN

Original BNC fresh biofilms from the cultures were examined using scanning electron microscopy (SEM, Carl Zeiss SUPRA 40 VP, Germany) at the Scientific Instrumentation Centre (CIC) of the University of Granada following the already described protocols [31]. Additionally, SEM (JSM-7800F, JEOL Ltd., Japan) at the ‘Servicio Central de Apoyo a la Investigación’ (SCAI, University of Córdoba, Spain), was used to observe purified and isolated UCO-Gly nanofibers. For this observation, the BNC suspension was initially homogenized using an Ultra-Turrax IKA T18 device (10,000 rpm, 4 min). Then, dropwise samples were deposited on glass covers (Ø = 10 mm) at a concentration of 0.01% wt. and allowed to air dry. Subsequently, the prepared samples were sputter-coated with gold according to previously described procedures [32]. Upon taking SEM images, ImageJ processing software for Windows (ver. 1.54g, National Institute of Health, Bethesda, MD, USA) was employed to measure the width and length of 100 randomly chosen fibers [33]. Finally, measurements were analyzed in OriginPro software for Windows (OriginPro 2018, ver. b9.5.1.195, OriginLab Corporation, Northampton, MA, USA) to determine the average size of the selected fibers [34].

#### 2.4.2. MorFi Analysis

The MorFi fiber analyzer (TECHPAP LB 01, Grenoble, France) was employed to examine UCO-Gly, BCNC-1, and BCNC-2 suspensions. Although this equipment is capable of measuring particles only in the micrometer scale, the resulting images provide a consistent indication of the effectiveness of the acid hydrolyses performed [35]. To perform this analysis, 40 mg of UCO-Gly were diluted in 1 L of distilled water and homogenized (Ultra-Turrax IKA T25 device, 9000 rpm, 4 min). In parallel, 10 mg of BCNC-1 and BCNC-2 were diluted in 1 L of distilled water and subsequently sonicated (7 min, 60% duty cycle, micro tip limit of 5). The homogenization of each cellulosic fragment was repeated under the same conditions just before continuous recirculation in the image acquisition system. Analyses were conducted for 5 min, with the size threshold for identifying fine particles set at 80 μm in length [36]. Additionally, the equipment provided the arithmetic mean of the length of each cellulosic form [37].

#### 2.4.3. Optical Microscopy

Microscopic examination of UCO-Gly and cellulosic fragments resulting from acid hydrolysis was performed to further confirm the success of the treatments. The analysis was carried out using an Axio Imager M1 optical microscope (Carl Zeiss, Oberkochen, Germany) equipped with an AxioCam MRc 5 digital camera. For UCO-Gly, a 0.1% wt. suspension was prepared in distilled water and homogenized (Ultra-Turrax IKA T25 device, 9000 rpm, 4 min). Likewise, BCNC-1 and BCNC-2 were diluted to a concentration of 10^−3^% wt. and subjected to ultrasonication (7 min, 60% duty cycle, micro tip limit of 5). Finally, a droplet of each resulting suspension was placed between a glass slide and a coverslip, and observations were made at a magnification of 10 × (numerical aperture = 0.25, focal length = 20 mm) [38]. Then, ImageJ processing software for Windows was employed to determine the macroscopic size (μm^2^) of BCNC-1 and BCNC-2 based on the methodology detailed by Desmaisons et al. (2017) [39]. Briefly, the software performed a comprehensive analysis of particles using the ‘analyze particles’ mode, which involved dividing the total surface area of the detected particles by the total count of particles in each image, resulting in the average size of the visible particles. Although six images were captured and further analyzed in both cases, only the most representative images are presented.

#### 2.4.4. Atomic Force Microscopy (AFM)

The morphology of produced BCNCs was examined via AFM (AFM Multimode—DI, Veeco, Instrumentation Group, Plainview, NY, USA). To conduct this analysis, three different suspensions of BCNC-1 and BCNC-2 with a concentration of 10^−4^% wt. in distilled water were prepared and homogenized via ultrasonication (7 min, duty cycle of 60%, micro tip limit of 5). Then, they were deposited dropwise on mica plates, allowing them to air dry overnight. AFM tests were conducted in tapping mode at room temperature. To ensure sample representativeness, at least 10 different areas of each sample were analyzed [40]. ImageJ software was used to determine both the length and width of the produced BCNCs, while size distributions were obtained using OriginPro software [34].

#### 2.4.5. Dynamic Light Scattering (DLS) and Zeta Potential (ZP)

To obtain an alternative perspective on the size distribution within the BCNC-1 and BCNC-2 populations, the average diameters were determined through dynamic light scattering using Zetasizer Pro (Malvern Panalytical, Worcestershire, UK) [41]. For this purpose, three dilutions of each BCNC sample were prepared in distilled water at a concentration of 0.1% wt. and loaded into disposable plastic cells, performing each test at room temperature. Each dilution was subjected to analysis 5 times, considering the mean value obtained through the equipment software (ZS XPLORER, ver. 3.2.1.11 for Windows, Malvern Panalytical Ltd., Worcestershire, UK) as the ‘particle size’ of each BCNCs population. In addition, the Zetasizer Pro was also employed to assess the surface charge of both BCNC-1 and BCNC-2 [42]. In brief, three dilutions of each BCNCs stock dispersion were prepared in distilled water to achieve a final concentration of 0.01% wt. These dilutions were then loaded into disposable folded capillary cells, and the analysis was performed in quintuplicate at room temperature. The average zeta potential value was also provided by ZS XPLORER software.

#### 2.4.6. X-Ray Diffraction (XRD)

The structure of UCO-Gly, BCNC-1, and BCNC-2 was analyzed using wide-angle XRD patterns [43]. All suspensions were first frozen (−20 °C, 24 h) and then lyophilized for 48 h at 0.01 mbar (ALPHA 2-4 LD plus, Christ^®^, Osterode am Harz, Germany). Each freeze-dried cellulosic material was then placed on the sample holder and leveled to ensure complete and uniform X-ray exposure. The samples were then examined on an X-ray diffractometer (PANalytical, The Netherlands) at room temperature using a monochromatic Cu Kα radiation source (radiation λ = 1.5419 Å). Scanning was performed in step-scan mode with a 2θ angle ranging from 5° to 60°, a step of 0.067°, and a scanning time of 5 min. The crystallinity index (*CrI*) of each cellulosic form was calculated according to Equation (1):

(1) $$CrI=\frac{\left(I_{002}-I_{am}\right)}{I_{002}}\times 100$$

where *I*_002_ represents the diffraction intensity of the main crystalline peak at 2θ ≈ 22.5° and *I_am_* is the intensity at 2θ ≈ 18° [44].

#### 2.4.7. Fourier Transform Infrared (FTIR) Spectroscopy

The FTIR spectra were acquired from freeze-dried samples of UCO-Gly, BCNC-1, and BCNC-2, employing PerkinElmer Spectrum 65 (PerkinElmer, Waltham, MA, USA). This method was used to investigate functional group shifts. The procedure was initiated with a background scan of air. Then, spectra were collected using the attenuated total reflectance (ATR) mode, covering the range from 4000 to 600 cm^−1^, with a resolution of 4 cm^−1^, and using 16 scans. A diamond ATR crystal was used, allowing for reliable surface analysis of the samples. Correction for the penetration depth (3 µm) was applied to account for the specific characteristics of the analyzed material. At least two spectra were obtained on different areas for each sample [45].

#### 2.4.8. Thermogravimetric Analysis (TGA)

The thermal properties of UCO-Gly, BCNC-1, and BCNC-2 samples were evaluated through TGA [46]. For this purpose, a small amount (10 mg approx.) of each freeze-dried cellulosic material was placed in ceramic alumina crucible capsules and subjected to a TGA/DSC 3+ device (Mettler Toledo, Greifensee, Switzerland) in a nitrogen atmosphere at a 20 mL/min flow rate. The temperature range was set from 25 to 800 °C, with a heating rate of 10 °C/min. The thermogravimetric analysis data and their derivative (DTG) were obtained using STARe-Evaluation software for Windows (ver. 16.40, Metter Toledo, Greifensee, Switzerland). In total, three samples were analyzed for this test.

### 2.5. Statistical Analysis

Statistical analyses of numerical data were conducted using SPSS software (ver. 25, IBM, New York, NY, USA) for Windows. One-way ANOVA and Tukey’s post hoc tests were performed to assess significant differences between groups. Results with a *p*-value ≤ 0.05 were considered statistically significant.

## 3. Results and Discussion

### 3.1. Bacterial Nanocellulose from Ancylobacter sp. STN1A

**Figure 1:**
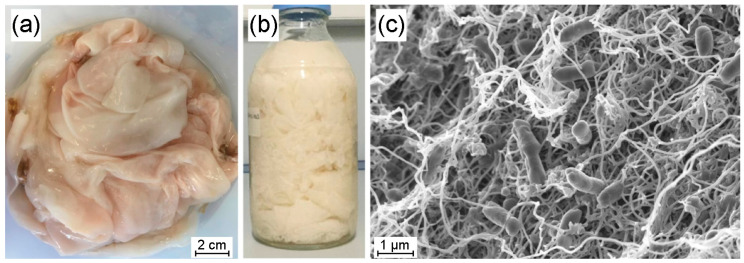
Fresh cellulose biofilms collected from the cultures before (**a**) and after the alkaline treatment in a 1 L bottle (**b**). Scanning electron microscopy (SEM) image of a sample of fresh biofilm before alkali treatment (**c**). The figure layout was designed using GIMP (MacOS, ver. 2.10.38).

The static cultivation of *Ancylobacter* sp. STN1A on the selected medium resulted in flat, smooth, and thick biofilms (Figure 1a,b), a morphology typically reported during BNC production [47]. SEM images of the original biofilms (Figure 1c) revealed an interwoven nanofiber network, a structure previously reported for BNC synthesized from crude glycerol via different Acetobacteriaceae species [48]. Furthermore, *Ancylobacter* sp. STN1A cells were observed within the network, showing a rod-like shape with an average length of 1.2 µm, consistent with other strains of this genus [49]. After alkaline treatment, washing, and drying, the average cellulose yield obtained was 2.20 ± 0.07 g/L. This value aligns with the range of productivity reported for bacterial strains commonly used in BNC synthesis with pure or crude glycerol as the sole carbon source [50]. According to Lee et al. (2021), impurities present in the UCO-Glycerine used (such as free fatty acids and glycerides, etc.), along with their metabolism by particular microorganisms, are key factors influencing the obtained productivity, which explains the variability in the previously reported values [51]. Given the growing industrial interest in this biomaterial, achieving competitive production yields becomes increasingly relevant. Indeed, recent technological advances have driven the BNC market to a current market valuation of approximately USD 300 million, with a projected growth of nearly 140% over the next seven years [52]. In parallel, the global biodiesel market is expected to reach nearly 50 billion liters by 2030, resulting in an estimated production of 5 billion liters of residual crude glycerol [19]. This surplus generation of glycerol poses significant environmental challenges related to its accumulation and management [53]. In this context, using residual crude glycerol as a raw material for BNC production would help to mitigate these environmental issues. At the same time, it would offer an economically viable alternative due to its favorable cost-to-productivity ratio, supporting a more sustainable approach aligned with circular economy principles.

### 3.2. SEM Characterization of UCO-Gly

**Figure 2:**
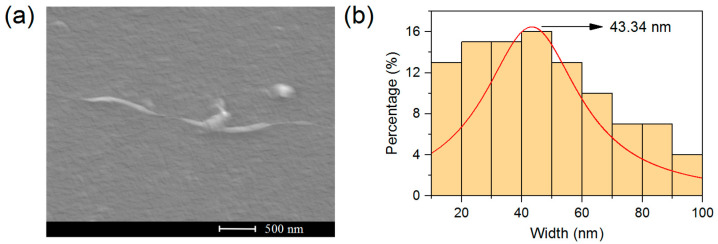
Results obtained from the SEM analysis of the BNC used in this work (UCO-Gly), showing the heterogeneity in width for both individual nanofibers (**a**) and the bulk sample (**b**). The average width value for the population was obtained after randomly analyzing 100 nanofibers using ImageJ and OriginPro software.

The morphological analysis of isolated nanofibers from the UCO-Gly biofilms (Figure 2) revealed substantial heterogeneity in their width, ranging from 10 to 100 nm. This variability is a well-documented characteristic of BNC production and can be attributed to biological factors such as bacterial density and nutrient concentration [54]. These variables can fluctuate during extended static incubation periods, contributing to the observed heterogeneity in fiber population. In any case, a population average value of approximately 43 nm was obtained for UCO-Gly width (Figure 2b), thus falling within the typical range for this biomaterial (30–50 nm) [55]. Indeed, one of the key characteristics of BNC compared to its plant-based counterpart is its smaller width and the absence of impurities [56]. Interestingly, the width value obtained for UCO-Gly was considerably lower than that reported by Gayathri and Srinikethan (2018), who also obtained BNC from crude glycerol metabolism, using a *Komagataeibacter saccharivorans* strain [48]. With regard to the length of UCO-Gly, it was difficult to obtain a representative value due to its considerable size, which hindered precise tracking in the images obtained. Nevertheless, it was possible to confirm its micrometer scale, in agreement with the results reported in the scientific literature [55]. This significant fiber length is probably related to the continuous cellulose synthesis during cell growth [57]. These results demonstrate the feasibility of using UCO-Gly for the synthesis of BCNCs with a high aspect ratio, expressed as the ratio of length to diameter (L/D) of these cellulose forms. This property generally leads to improved mechanical properties when BCNCs are incorporated into nanocomposites, as suggested by previous research [58].

### 3.3. MorFi Evaluation

**Figure 3:**
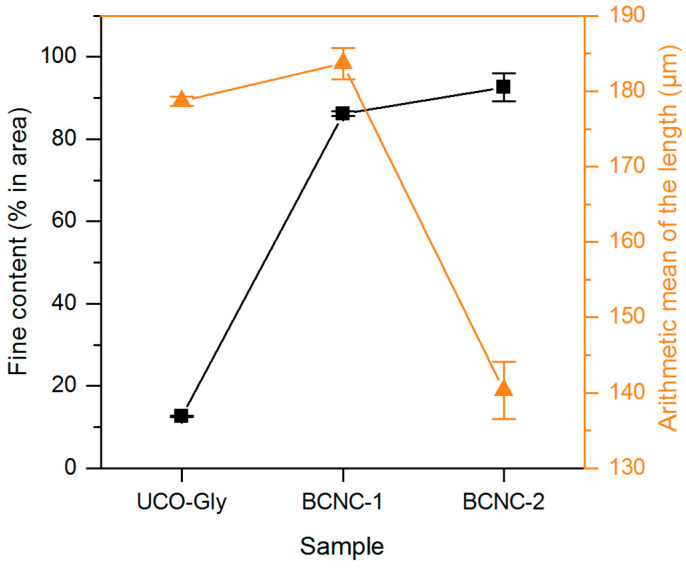
Morphological analysis via MorFi of pristine BNC (UCO-Gly) and nanocellulosic forms obtained after severe (BCNC-1) and mild (BCNC-2) acid hydrolysis. The figure shows the fine content (% in area) and the arithmetic mean of fiber length (μm) measured by the equipment for each sample, illustrating the impact of the applied treatments.

MorFi analysis tool offers an alternative nanoscale approach, efficiently monitoring the effects of the applied treatments on cellulose samples. These comprehensive insights into fiber populations are valuable in tailoring treatments for nanofibers [59]. As shown in Figure 3, BCNC-1 and BCNC-2 exhibited significant increases (*p* < 0.05) in fine content, rising by 583% and 634% compared to UCO-Gly, respectively. These findings underscore the impact of acid hydrolysis on cellulose biomass, resulting in the disruption of large aggregates and a subsequent rise in the number of smaller particles in the sample [35]. Regarding the arithmetic average value for fiber length, BCNC-1 exhibited nearly identical values to the starting material (*p* > 0.05). This alignment and the greater fine content in BCNC-2 than in BCNC-1 agree with previous research [35]. Indeed, as the sample size decreases, the measurement equipment reaches a threshold where detecting the targeted structures becomes unfeasible. According to Pennells et al. (2022), the theoretical detection limit of the MorFi equipment is 5 µm for the fine element length, meaning that nanocellulosic forms below this threshold are not captured by the device [59]. Additionally, BCNC-2 exhibited a significant 21% reduction in fiber length compared to the starting nanofibers, also aligning with previous research using MorFi to evaluate other cellulose fiber fragmentation methods [36]. These observations suggest that while both treatments effectively broke down UCO-Gly, the more severe hydrolysis conditions (i.e., BCNC-1) led to smaller cellulose fragments. This highlights the importance of adjusting the hydrolysis conditions to achieve the desired fragmentation for specific applications.

### 3.4. Optical Microscopy Evaluation

**Figure 4:**
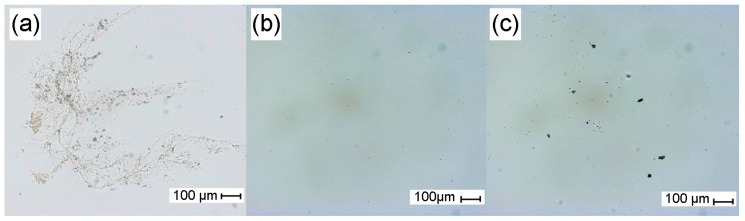
Optical microscopy images of UCO-Gly (**a**), BCNC-1 (**b**), and BCNC-2 (**c**) suspensions, obtained in bright-field mode with transmitted light. The figure layout was designed using GIMP (MacOS, ver. 2.10.38).

The most representative images resulting from the microscopic observation of each sample are shown in Figure 4. UCO-Gly suspension was characterized by a generally homogeneous appearance, occasionally featuring bundles of entangled nanofibers that were visible at the micrometer scale (up to 2 mm in length, 4–6 μm in width), as shown in Figure 4a. These entanglements are common when examining cellulose fibers under optical microscopy [38]. In our case, the obtained values are directly linked to the substantial length inherent to BNC, explaining their increased tendency to form larger aggregates in suspension [55]. As expected, none of these structures were observed in BCNC-1 and BCNC-2 (Figure 4b and Figure 4c, respectively). After using the ratio of the total visible surface area of the cellulose particles to their count (see Section 2.4.3), the ImageJ software determined a macroscopic size of 6.00 ± 0.19 μm^2^ for BCNC-1. In contrast, BCNC-2 exhibited a significantly larger (*p* < 0.05) value of 13.18 ± 3.22 μm^2^. This difference is attributed to the less severe acid hydrolysis conditions applied for BCNC-2, resulting in the generation of larger cellulose fragments compared to BCNC-1, which were even visible under the light microscope (Figure 4c) [60]. Both findings are consistent with the MorFi analysis, indicating that the equipment was unable to detect the cellulose fragments produced in BCNC-1.

### 3.5. AFM Characterization of BCNC-1 and BCNC-2

**Figure 5:**
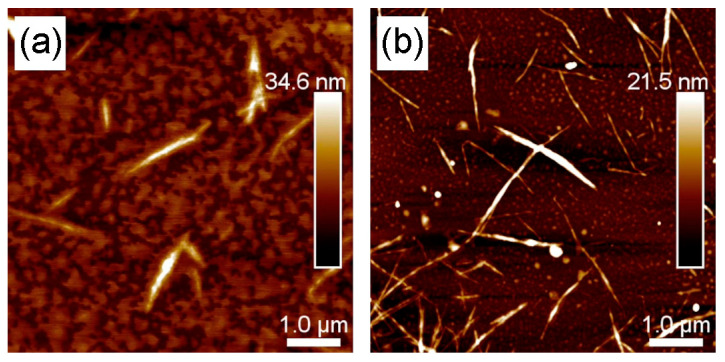
Atomic force microscopy images of BCNC-1 (**a**) and BCNC-2 (**b**) samples. The figure layout was designed using GIMP (MacOS, ver. 2.10.38).

The results discussed above were further supported by AFM observations. Indeed, both acid hydrolysis treatments applied to UCO-Gly successfully led to the formation of well-defined cellulose nanocrystals, although they exhibited notable variations in morphology (Figure 5). BCNC-1 images (Figure 5a) revealed an average population length of 913 nm and a width of 40 nm, resulting in an aspect ratio (L/D) of 22.8. In contrast, BCNC-2 (Figure 5b) exhibited a significantly higher (*p* < 0.05) average length of 1130 nm and a slightly greater average width of 42 nm, resulting in an L/D of 26.9. These results indicate that longer acid hydrolysis treatment produces shorter and narrower nanocrystals, thus agreeing with previous research [61]. However, the obtained length and width for both BCNCs populations were significantly greater than those documented in prior studies with comparable treatments of BNC [14]. This emphasizes the role of both the bacterial strain and the different factors discussed earlier (see Section 3.2) in shaping the morphology of the synthesized BNC and, consequently, in the structure of the resulting BCNCs. Regarding the aspect ratio, it is noteworthy that the value obtained for BCNC-2 is significantly higher than that reported in previous studies of BNC hydrolysis for the same time but with a lower H_2_SO_4_ concentration, further supporting earlier findings [22]. The L/D value of CNCs has been demonstrated to be a key parameter in modifying the mechanical and barrier properties of different polymeric matrices [62]. Therefore, the values obtained in this work highlight the great potential of the produced BCNCs for reinforcing functional materials [7]. Indeed, several researchers are actively investigating this issue [63,64,65].

### 3.6. DLS and Zeta Potential

**Figure 6:**
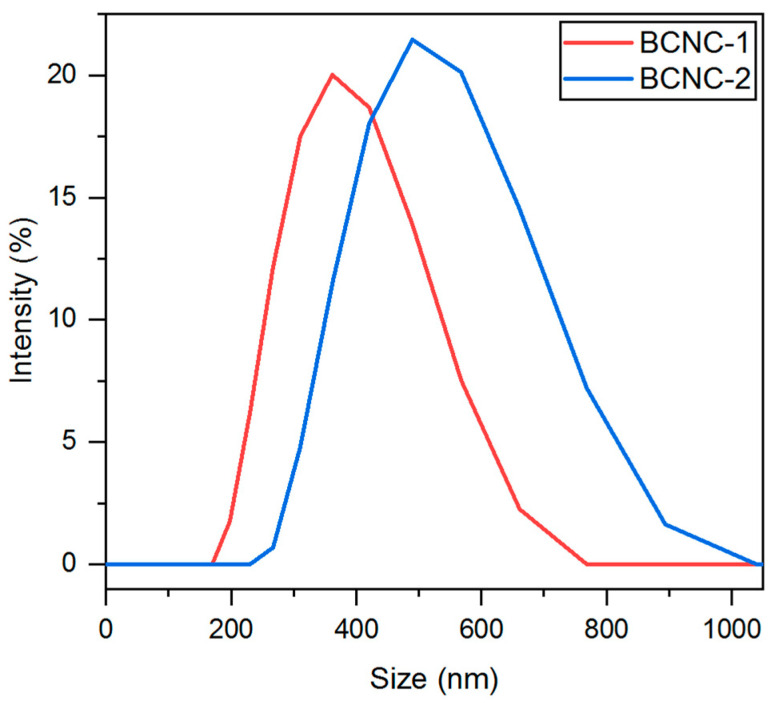
Particle size distribution profiles derived from dynamic light scattering (DLS) analysis for BCNC-1 and BCNC-2.

DLS is commonly employed to assess the main size distribution range of non-spherical nanoparticles, such as CNCs. However, as it measures the hydrodynamic radius, its value often deviates from measurements obtained through other observation techniques. Hence, DLS is more often used to establish relative differences between particles subjected to the same treatment [66,67,68]. As shown in Figure 6, the values obtained for BCNC-1 ranged from 169 to 766 nm, whereas BCNC-2 exhibited higher dimensions, spanning from 230 to 1037 nm. These results align with previous research, associating more severe acid hydrolysis conditions (i.e., BCNC-1) with a reduction in the DLS size (also known as apparent size) of the obtained nanocrystals [60]. Additionally, these values are consistent with the results obtained in AFM characterization, which evidenced nanocrystals with increased length and width when milder acid hydrolysis conditions were applied (i.e., BCNC-2). Moreover, a single set of measurements was obtained in both cases, indicating that the acid hydrolysis led to the formation of uniform BCNCs populations.

In addition, the obtained ZP values were used to evaluate the suspension stability of BCNC-1 and BCNC-2. According to the literature, ZP values ranging from −15 to 15 mV indicate a low surface charge. This lack of charge is insufficient to promote repulsion, which results in the aggregation of BCNCs. In contrast, values below −30 mV or exceeding 25–30 mV characterize stable suspensions in water [69]. In this work, ZP values of −40.17 ± 0.68 mV and −44.13 ± 0.73 mV were obtained for BCNC-1 (Figure S1 of the Supplementary Materials) and BCNC-2 (Figure S2 of the Supplementary Materials), respectively, thus agreeing with the range of values reported in similar previous studies [70]. These values indicate that the acid hydrolysis treatment successfully led to the formation of a negative electrostatic layer on the surface of both BCNCs, achieved through the grafting of sulfate groups (–OSO_3_^−^) via the esterification of hydroxyl (–OH) groups. Moreover, the significantly lower (*p* < 0.05) value obtained for BCNC-1 is attributed to the fact that severe reaction conditions directly impact the structure and size of the nanocrystals, as demonstrated earlier in this study and supported by other researchers [14]. Therefore, the ZP values obtained in this work are more negative than those typically reported for acid hydrolysis-derived cellulose nanocrystals from plant sources [71,72]. This can be attributed to an enhanced total surface area available for sulfate group grafting on BCNCs. Overall, the observed size uniformity in both BCNCs and their remarkable colloidal stability also indicate promising potential for their application as material reinforcement agents [69].

### 3.7. XRD Analysis

**Figure 7:**
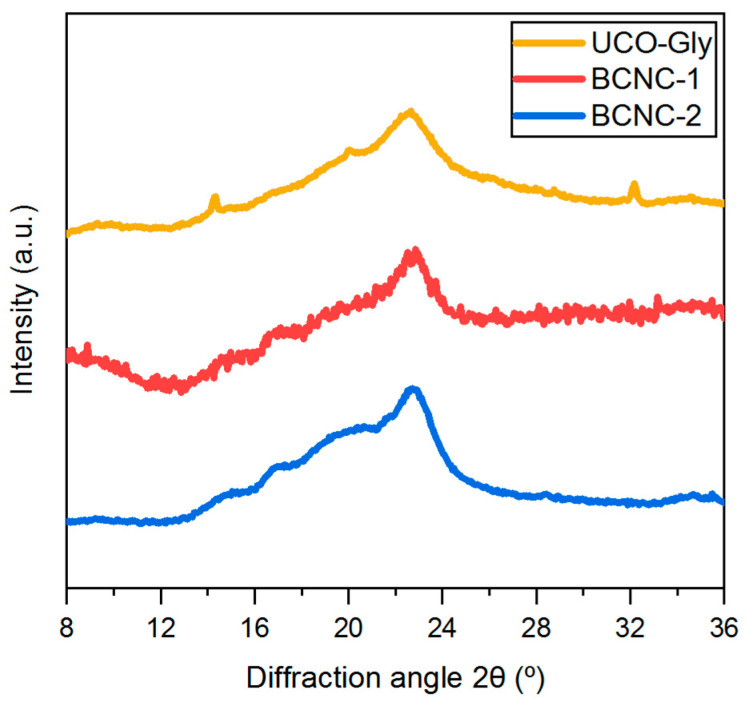
X-ray diffraction (XRD) patterns of UCO-Gly, BCNC-1, and BCNC-2, displaying the typical diffraction peaks associated with the allomorphic cellulose I structure. This arrangement was preserved across treatments, regardless of whether severe (BCNC-1) or mild (BCNC-2) acid hydrolysis was applied.

The obtained XRD patterns (Figure 7) showed remarkable similarities between UCO-Gly and BCNC-2, although both exhibited subtle differences compared to BCNC-1. Indeed, characteristic peaks at 14.33°, 22.61°, and 34.62° were identified in UCO-Gly, along with a smoother peak at 16.79°, indicative of the typical allomorphic structure of cellulose I [73]. Additionally, a peak at 20.33° characteristic of the monoclinic structure of cellulose II was observed. This conversion from cellulose I to cellulose II may have occurred during the initial washing of the membranes with NaOH solution, which resulted in cellulose mercerization [74]. As mentioned above, these peaks were also observed in the pattern obtained for BCNC-2, indicating a fairly similar structure between both cellulose forms. Likewise, UCO-Gly and BCNC-2 showed comparable CrI values of 53% and 55%, respectively. The CrI value obtained for UCO-Gly was notably lower than those reported in previous related works [51,74]. This highlights the influence of the bacterial strain used on the structure of the synthesized biomaterial, suggesting potential variations in its properties and performance for those applications requiring tailored mechanical or physicochemical characteristics. Conversely, BCNC-1 exhibited a considerably higher CrI value of 63%. This notable discrepancy is directly linked to the nature of bacterial cellulose nanofibers; BNC usually needs more reaction time to break down their bundles and remove the amorphous regions [61]. Moreover, only the characteristic peaks of cellulose I were observed on its pattern, representing the crystal structure with the highest axial elastic modulus [75]. Considering these findings, BCNC-1 would be suitable for incorporation into composite materials requiring a high axial elastic modulus and a more defined crystalline structure, such as those used in the aerospace or automotive industries [76].

### 3.8. FTIR Characterization

**Figure 8:**
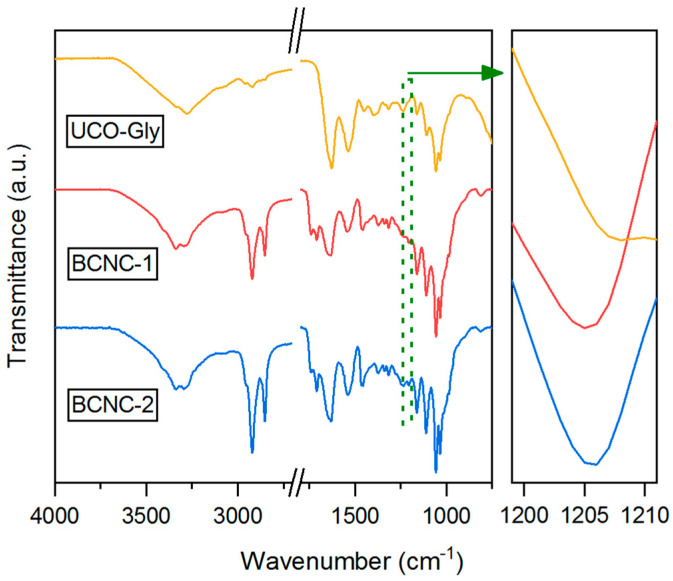
Fourier transform infrared (FTIR) spectra in transmittance mode of each cellulosic form analyzed in this work.

The FTIR spectra obtained for UCO-Gly, BCNC-1, and BCNC-2 (Figure 8) were highly similar, indicating an identical chemical composition among the three cellulose forms analyzed [77]. Indeed, all of them exhibited typical absorption peaks around 3000–3650 cm^−1^ (O–H stretching of intramolecular hydrogen bonds), 2920 cm^−1^ and 2853 cm^−1^ (–CH_2_ stretching), 1430 cm^−1^ (CH_2_ symmetric bending), 1371 cm^−1^ (symmetric angular deformation of C–H bonds), 1160 cm^−1^ (asymmetrical stretching of C–O–C glycoside bonds), 1110 cm^−1^ (stretching of C–OH and C–C–OH bonds in secondary alcohols), and 1059 cm^−1^ (stretching of C–OH and C–C–OH bonds in primary alcohols) [14,61,78]. However, subtle changes were observed in some of the absorption peaks in both BCNC spectra. In fact, the region from 3650 to 3000 cm^−1^ appeared slightly modified in intensity and shape, suggesting a change in the H-bonding pattern due to the acid hydrolysis treatment. Additionally, a new absorption peak at 1740 cm^−1^ (bending vibration of –COOH) was observed, indicating further oxidation of the pyranose ring [15]. Similarly, a new peak around 1205 cm^−1^ was identified, indicative of the substitution of –OH groups by sulfate groups during the reaction [22]. In this regard, the characteristic absorption peak of the esterification of hydroxyl groups in cellulose chains (symmetric vibration of C–O–S bonds associated with C–O–SO_3_^−^ groups) was also observed around 813 cm^−1^ [14]. Furthermore, it was observed that the absorption peak around 1430 cm^−1^, commonly referred to as the ‘crystallinity band’, exhibited greater intensity in both BCNCs samples compared to UCO-Gly. This aligns with the results obtained from XRD analysis, thereby confirming the effectiveness of the hydrolysis treatment in breaking down the amorphous regions of UCO-Gly [30].

### 3.9. Thermal Properties

**Figure 9:**
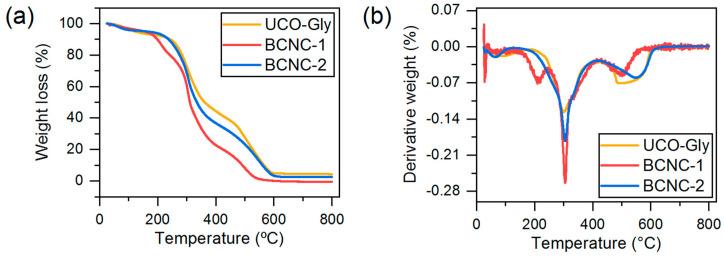
Thermogravimetric analysis (**a**) and derivative thermogravimetry (**b**) profiles obtained for UCO-Gly and the produced BCNC-1 and BCNC-2.

The mass loss profiles obtained for the three analyzed cellulose forms are shown in Figure 9a. The three typical events during the thermal decomposition of BNC and its derived BCNCs were identified (Figure 9b). Specifically, a first slight mass loss at 25–140 °C was observed, corresponding to the evaporation of water associated with the cellulosic material. A second stage of mass loss, between 180 and 420 °C, is associated with the dehydration, decomposition, and depolymerization of the glucoside units. Finally, a third step from 450 to 600 °C was detected, where the oxidation and decomposition of the carbonaceous residues occur [79]. Regarding the differences observed between the degradation curves, it was noted that the main degradation step was shifted towards lower temperatures of approximately 65 °C for BCNC-1 and around 35 °C for BCNC-2 compared to UCO-Gly. This is directly related to the substitution of cellulose –OH groups by sulfate groups during acid hydrolysis treatment, as well as the larger surface area present in both types of BCNCs. This functional group substitution reduces the activation energy required for the thermal degradation catalysis of BCNC-1 and BCNC-2, indicating reduced thermal stability, as demonstrated by other authors and consistent with the results presented above in this study [22,80]. Interestingly, the degradation profile obtained for BCNC-1 showed an additional degradation event within the main degradation stage, with an onset temperature of 180 °C, which is related to the degradation of regions associated with sulfate groups [81]. However, this event was not present in the degradation profile obtained for BCNC-2, indicating the lower sulfation of –OH groups due to the shorter reaction time. This would also explain the similar thermal stability obtained for BCNC-2 and UCO-Gly. On the other hand, a shift in the T_max_ of degradation from 300 to 305 °C was observed in both forms of BCNCs compared to UCO-Gly. This suggests the presence of a higher crystalline fraction, more resistant to acid hydrolysis treatment, for these cellulosic forms [14]. Finally, in the final segment of the TGA degradation pattern, the high purity of the starting cellulose sample used, as well as of the subsequently produced BCNCs, was demonstrated. Indeed, residual masses of 4.35%, 0%, and 2.62% were obtained for UCO-Gly, BCNC-1, and BCNC-2, respectively. These values are considerably lower than those reported in acid hydrolysis studies of plant cellulose nanofibers, due to the thermoresistant components generally associated with them [72,82]. Overall, understanding these thermal degradation patterns is essential for optimizing manufacturing processes. This ensures the desired performance of composite materials where BCNCs could be used as nanofillers, such as in polymeric materials, the paper industry, or food sector [83].

## 4. Conclusions

This work presented a novel pathway for obtaining bacterial cellulose nanocrystals (BCNCs) through the valorization of residual crude glycerol. For this, *Ancylobacter* sp. STN1A was used for BNC biosynthesis, a bacterial strain that has not been previously applied for this process. The acid hydrolysis conditions applied to BNC were shown to modulate the morphological, structural, and thermal features of the resulting BCNCs, enabling the generation of nanocellulosic forms with tunable properties. These findings not only reinforce the versatility of BCNCs as customizable materials, but also demonstrate the feasibility of integrating waste valorization into nanomaterial synthesis. Indeed, the use of residual crude glycerol enables the cost-efficient production of these bio-based materials, while also contributing to waste reduction in the biodiesel industry, thus aligning with circular economy principles. Future research could focus on adjusting the extraction process, evaluating the functional performance in specific applications (such as biomedicine, environmental remediation, or sustainable packaging, among others), and assessing the scalability of this approach to enable its industrial implementation. Overall, this study offers both scientific and technological insights that support the advancement of sustainable nanomaterials from underutilized industrial byproducts.

## Data Availability

The raw data supporting the conclusions of this article will be made available by the authors upon request.

## Supplementary Materials

The following supporting information can be downloaded at: https://www.mdpi.com/article/10.3390/polym17091240/s1. Figure S1: representative zeta potential distribution obtained for BCNC-1; Figure S2: representative zeta potential distribution obtained for BCNC-2.

## Abbreviations

The following abbreviations are used in this manuscript:

NFC	Nanofibrillated cellulose
CNCs	Cellulose nanocrystals
BNC	Bacterial nanocellulose
BCNCs	Bacterial cellulose nanocrystals
UCO-Glycerine	Used cooking oil-derived crude glycerine
WPM	Widdel production medium
WMM	Widdel mineral medium
SEM	Scanning electron microscopy
AFM	Atomic force microscopy
DLS	Dynamic light scattering
ZP	Zeta potential
XRD	X-ray diffraction
FTIR	Fourier transform infrared
ATR	Attenuated total reflectance
TGA	Thermogravimetric analysis
DTG	Derivative thermogravimetry
