# Supplementary material for: Isolation and Characterization of Cellulose Nanocrystals from Bacterial Cellulose Synthesized via Ancylobacter sp. STN1A Using Residual Glycerol
Source: Polymers (Basel). 2025 May 1;17(9):1240. doi: 10.3390/polym17091240 (PMC12073757; doi:10.3390/polym17091240)
Supplement: Supplementary file 1 [file polymers-17-01240-s001.zip › polymers-3608635-supplementary.pdf]

Supplementary materials

Isolation and Characterization of Cellulose Nanocrystals from Bacterial Cellulose Synthesized via *Ancylobacter* sp. STN1A Using Residual Glycerol

Manuel Peña-Ortiz[1,2,3], Araceli García[1,3], Sophie Marie Martirani-Von Abercron[4], Patricia Marín[4], Silvia Marqués[4], Ramzi Khiari[5,6], Alain Dufresne[6], Luis Serrano[2,3*]

[1] Organic Chemistry Department, Nanoval FQM-383 Research Group, University of Córdoba, Marie Curie (C-3) Building, Ctra. Nnal. Km 396, 14014 Córdoba, Spain; b52penom@uco.es (M.P.-O.); qo2ganua@uco.es (A.G.)
[2] Inorganic Chemistry and Chemical Engineering Department, BioPrEn RNM 940 Research Group, University of Córdoba, Marie Curie (C-3) Building, Ctra. Nnal. Km 396, 14014 Córdoba, Spain; b52penom@uco.es (M.P.-O.); iq3secal@uco.es (L.S.)
[3] Faculty of Science, Instituto Químico Para la Energía y el Medioambiente (IQUEMA), University of Córdoba, Marie Curie (C-3) Building, Ctra. Nnal. Km 396, 14014 Córdoba, Spain; b52penom@uco.es (M.P.-O.); qo2ganua@uco.es (A.G.); iq3secal@uco.es (L.S.)
[4] Estación Experimental del Zaidín, Department of Biotechnology and Environmental Protection, Consejo Superior de Investigaciones Científicas, C/. Profesor Albareda 1, 18008, Granada, Spain; sophie.martirani@eez.csic.es (S.M.M.-V.A.); pmarin@eez.csic.es (P.M.); silvia.marques@eez.csic.es (S.M.)
[5] Department of Textile, Higher Institute of Technological Studies of Ksar Hellal, Ksar Hellal 5070, Tunisia; ramzi.khiari@grenoble-inp.fr (R.K.)
[6] Université Grenoble Alpes, Centre National de la Recherche Scientifique (CNRS), Grenoble INP, Laboratory of process engineering for biorefinery, bio-based materials and functional printing (LGP2), F-38000 Grenoble, France; alain.dufresne@pagora.grenoble-inp.fr (A.D.)
* Correspondence: iq3secal@uco.es; Tel.: +34 957218556

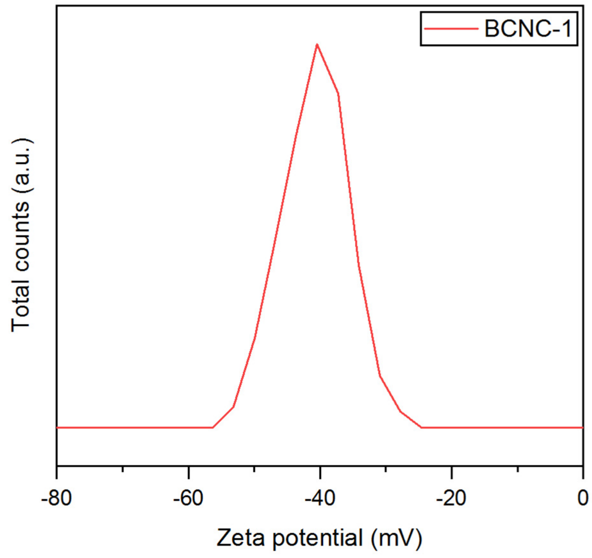

Figure S1. Representative zeta potential (ZP) distribution obtained for BCNC-1.

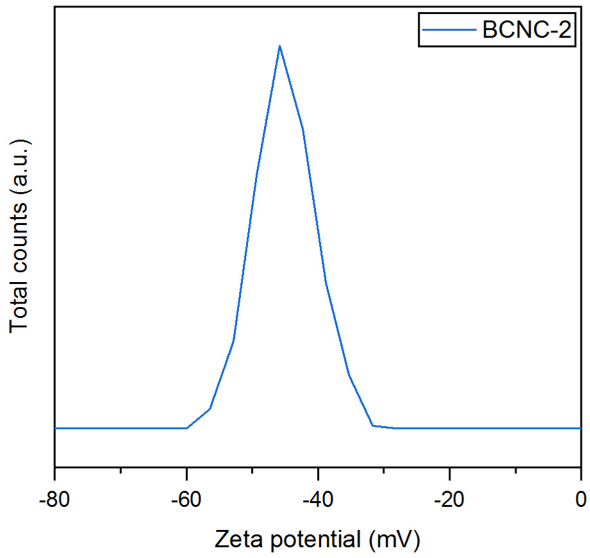

Figure S2. Representative zeta potential (ZP) distribution obtained for BCNC-2.
